# Fire Performance of FRP-RC Flexural Members: A Numerical Study

**DOI:** 10.3390/polym14020346

**Published:** 2022-01-17

**Authors:** Dexin Duan, Lijun Ouyang, Wanyang Gao, Qingfeng Xu, Weidong Liu, Jian Yang

**Affiliations:** 1 State Key Laboratory of Ocean Engineering, Shanghai Jiao Tong University, Shanghai 200240, China; duan_dexin@sjtu.edu.cn (D.D.); j.yang.1@sjtu.edu.cn (J.Y.); 2 Shanghai Key Laboratory of Engineering Structure Safety, Shanghai Research Institute of Building Sciences Co., Ltd., Shanghai 200032, China; xuqingfeng73@163.com; 3 School of Environment and Architecture, University of Shanghai for Science and Technology, Shanghai 200093, China; wdliu2010@126.com; 4 Shanghai Key Laboratory for Digital Maintenance of Buildings and Infrastructure, School of Naval Architecture, Ocean and Civil Engineering, Shanghai Jiao Tong University, Shanghai 200240, China

**Keywords:** flexural members, FRP bar, fire performance, bond degradations, high temperatures

## Abstract

Fiber-reinforced polymer (FRP) bars are increasingly used as a substitute for steel reinforcements in the construction of concrete structures, mainly due to their excellent durability characteristics. When FRP bar-reinforced concrete (referred to as FRP-RC for simplicity) members are used in indoor applications (e.g., in buildings), the fire performance of FRP-RC members needs to be appropriately designed to satisfy safety requirements. The bond behavior between the FRP bar and the surrounding concrete governs the composite action between the two materials and the related structural performance of the FRP-RC flexural member that will be affected when exposed to fire. However, there is a lack of reliable numerical models in the literature to quantify the effect of bond degradations of the FRP bar-to-concrete interface at high temperatures on the fire performance of FRP-RC flexural members. This paper presents a three-dimensional (3D) finite element (FE) model of FRP-RC flexural members exposed to fire and appropriately considers the temperature-dependent bond degradations of the FRP bar-to-concrete interface at high temperatures. In addition, the thermal properties of concrete and FRP bars are considered in the heat transfer analysis to predict the cross-sectional temperatures of the FRP-RC members under fire exposure. In the FE model, the mechanical properties and constitutive laws of concrete and FRP bars at high temperatures in addition to the bond degradations between them have been properly defined, thereby accurately predicting the global and local structural responses of the FRP-RC members under fire exposure. The proposed FE model has been validated by comparing the FE predictions (both temperature and midspan deflection responses during fire exposure) and the full-scale fire test results reported in the literature. The validated FE model is then used to study the effects of bond degradations on the global and local structural responses of the FRP-RC members under fire exposure. It is proved that the temperature-dependent bond degradations need to be considered to achieve accurate predictions of the failure mode and deflection responses.

## 1. Introduction

Fiber-reinforced polymer (FRP) bars are increasingly used as a substitute for steel reinforcements in the construction of concrete structures, mainly due to their excellent durability properties. In the literature, a large number of studies have been conducted on the performance of FRP bar-reinforced concrete (referred to as FRP-RC for simplicity) members at ambient temperature [1,2,3,4], and the related design provisions have been specified in the current design guidelines [5,6]. However, the FRP-RC members are likely to be exposed to fire hazards during their service life, especially when these members are used in indoor applications (e.g., in buildings) [7,8,9,10]. Under high-temperature exposure in a fire, the material properties of FRP bars and concrete as well as the bond behavior between them will usually be significantly reduced [11,12], possibly leading to a significant reduction in the load-carrying capacity of the FRP-RC members [13,14]. Therefore, fire performance of the FRP-RC members is an essential issue in the design process and should be properly considered to meet the requirements specified in the current design guidelines [5,6].

Existing studies in the literature mainly focus on the mechanical and bond properties of externally bonded FRP laminates at high temperatures [15,16,17,18,19,20,21,22,23,24,25,26,27,28] and the related fire performance evaluation of FRP-strengthened RC members [29,30,31,32,33,34,35,36,37,38,39,40,41], while relatively limited information is available on the fire performance of FRP-RC members. A number of fire tests have been carried out on full-scale FRP-RC flexural members under standard fire exposure conditions [42,43,44,45,46]. The test results have indicated that if the FRP-RC member is properly designed (such as using a thick concrete cover and/or well-protected anchorage zone), it can achieve a satisfactory fire-resistance rating. Moreover, it is generally believed that the fire resistance period of the FRP-RC flexural member is lower than that of the corresponding conventional steel-RC member. This is because compared with steel bars, the FRP bars exhibit more significant reductions in the mechanical and bond properties at high temperatures. Apart from the fire tests, some numerical or finite element (FE) models are also proposed in the literature to predict the fire performance of FRP-RC flexural members [14,47,48,49,50,51]. The numerical and test results provided in the existing literature have promoted a good understanding of the thermal and structural responses of FRP-RC flexural members under fire exposure [51,52].

It is noteworthy that under fire exposure, high thermal stresses may be generated at the interface between the FRP bar and the surrounding concrete cover, mainly due to the different thermal expansion coefficients between FRP and concrete. When the thermal stress increases to the tensile strength of the concrete cover during fire exposure, it will be partially cracked, resulting in the weakening of the bond between FRP and concrete [53,54]. Moreover, the existing literature has indicated that the bond strength degradations between the FRP bars and the surrounding concrete at high temperatures are more severe than the mechanical property reductions of the FRP bars [11,55,56,57]. Therefore, the bond behavior between the FRP bars and the concrete plays a crucial role in governing the load transfer between FRP and concrete as well as the structural responses and fire performance of FRP-RC flexural members [58,59]. However, almost all existing numerical and FE studies [14,47,48,49,50,51] neglect the influence of bond degradations at high temperatures on the fire performance of FRP-RC flexural members. The proposed FE model is expected to act as a reliable computational tool for the parametric study of FRP-RC flexural members under fire exposure. Based on the parametric study results, a rational design method will be established for the fire resistance design of FRP-RC flexural members in the future.

Given the above research background, this paper has proposed a three-dimensional (3D) FE model to study the effect of bond degradations of the FRP bar-to-concrete interface at high temperatures on the fire performance of FRP-RC flexural members. The FE model has appropriately considered the constitutive laws of FRP and concrete as well as their bond interface at high temperatures, and therefore, it is expected to accurately predict the thermal and structural responses of FRP-RC flexural members under fire exposure. Then, the proposed FE model is verified by comparing the FE results with the test data of the full-scale fire tests in the literature. The validated FE model is further used to study the effect of the interfacial bond degradations at high temperatures on the fire performance of FRP-RC flexural members. The results have indicated that the consideration of the bond degradations of the FRP bar-to-concrete interface can capture the pull-out failure mode of the FRP bars and yields more accurate predictions of the deflection responses of FRP-RC flexural members under fire exposure.

## 2. Procedure of the FE Model

### 2.1. Modeling Procedure

The modeling procedure of the FRP-RC members in fire includes two main steps: heat transfer analysis and mechanical analysis. Heat transfer analysis is used to determine the cross-sectional temperature distributions of the FRP-RC flexural member under fire exposure, while the mechanical analysis takes the cross-sectional temperature distributions as the initial state to determine the structural responses. In other words, the modeling procedure follows a sequential thermo-mechanical analysis in which it is assumed that the results of the mechanical analysis have no effect on the heat transfer analysis of the same FRP-RC member under fire exposure. This sequential thermo-mechanical analysis was also successfully adopted by the corresponding author for predicting the fire performance of RC beams and insulated FRP-strengthened RC beams [39,60,61]. The following subsections give the details of the thermal properties of concrete and FRP bars and the boundary conditions considered in the heat transfer analysis, while the modeling of the temperature-dependent constitutive laws of the material and bond properties of concrete and FRP bars at ambient and high temperatures for the mechanical analysis will be provided in detail in the next sections.

### 2.2. Thermal Properties of Concrete and FRP Bars

**Figure 1:**
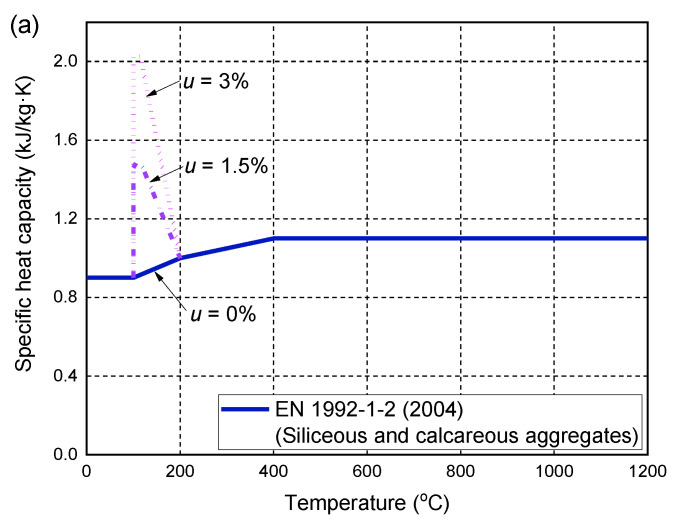

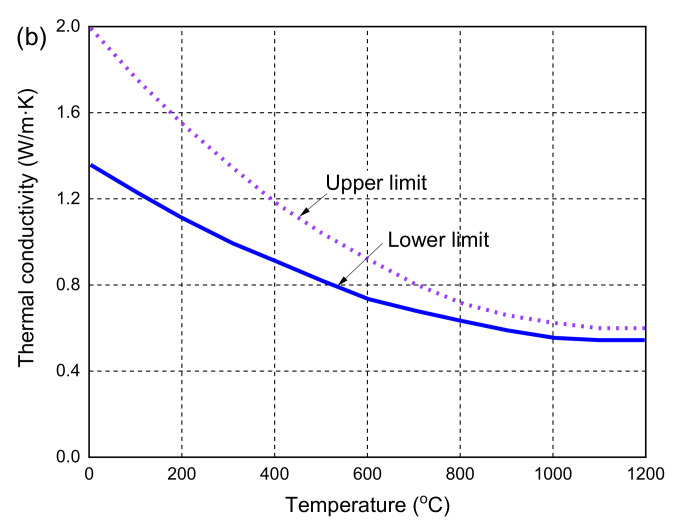
Thermal properties of concrete at high temperatures: (**a**) specific heat capacity; (**b**) thermal conductivity.

The thermal properties of concrete have been extensively studied in the literature, including thermal conductivity, specific heat capacity, and density at high temperatures. The temperature-dependent changes of these parameters are provided in current design guidelines, such as EN 1992-1-2 [62]. It provides design formulas for the specific heat capacity of concrete made of siliceous and calcareous aggregates at high temperatures (Figure 1a). By considering the heat absorption of water evaporation, the increase in the specific heat is considered at 100–115 °C, depending on the moisture content of the concrete. Figure 1a shows the changes in the peak specific heat capacity of concrete with different moisture contents of 0, 1.5, and 3%. For other moisture contents, linear interpolation can be used to calculate the peak value. Figure 1b shows the upper and lower bound limits of the thermal conductivity of ordinary concrete at ambient and high temperatures. In this study, each of the lower and upper bound limits has been incorporated into the heat transfer analysis to determine which one is more accurate to achieve a closer agreement with the fire test results. Through a trial-and-error analysis, the lower bound limit usually yields more accurate temperature predictions of FRP-RC flexural members under fire exposure. More details of the temperature predictions are given in Section 6. A similar method was also adopted by Hajiloo and Green [47] to obtain a reliable definition of the thermal conductivity of concrete at high temperatures. The density of concrete is considered to be 2300 kg/m^3^, which is usually assumed to be constant at high temperatures [47,61].

Limit information is available on the thermal properties of FRP composites at high temperatures. In the heat transfer analysis, the temperature-dependent thermal properties (i.e., thermal conductivity, specific heat capacity, and density) of FRP bars are determined based on the experimental results reported by Griffis et al. [63]. Such thermal properties of FRP composites were also used in previous numerical and FE studies to predict the temperature responses of RC members with externally bonded or internally reinforced FRP reinforcements [29,39,41,47].

### 2.3. Definitions of the Boundary Conditions for Heat Transfer Analysis

The heat exchange between the hot fluxes of fire and the surfaces of the FRP-RC member is realized through heat convection and heat radiation, which is controlled by the Robin boundary conditions as follows:

(1) $$-k\frac{\delta T}{\delta n}=q_{c}+q_{r}$$

where k is the thermal conductivity, n is the normal direction of the surface, and $q_{c}$ and $q_{r}$ are the heat fluxes produced by heat convection and heat radiation, respectively, which can be described as follows:

(2) $$q_{c}=h_{c}\left(T-T_{f}\right)$$

(3) $$q_{r}=\varepsilon_{m}\varepsilon_{f}\sigma \left[{\left(T-T_{z}\right)}^{4}-{\left(T_{f}-T_{z}\right)}^{4}\right]$$

where $h_{c}$ is the convective heat transfer coefficient, $h_{c}$ = 25 W/(m^2^·K) is used for the surfaces of the FRP-RC member exposed to fire, and $h_{c}$ = 9 W/(m^2^·K) for the unexposed surface [64,65]. $\varepsilon_{m}$ represents the thermal emissivity of the member surface, and $\varepsilon_{f}$ represents that of the hot fluxes of fire, which are determined to be 0.8 for concrete and 1.0 for the standard fire conditions as per EN 1991-1-2 [64]. $T_{f}$ is the hot flux temperature of the fire, T is the surface temperature of the FRP-RC member, and $T_{z}$ is the absolute zero temperature. σ is the Stefan-Boltzmann constant and is equal to 5.67 × 10^−8^ W/(m^2^·K^4^).

The heat transfer within the FRP-RC member is realized by heat conduction, which is described by the following equation:

(4) $$k\nabla^{2}T-\rho c\frac{\delta T}{\delta t}=0$$

where ρ and c are the density and specific heat capacity, respectively, and t is the fire-exposure time. The solution of Equation (4) can be obtained by the above boundary conditions and initial temperature distribution. The latter at *t* = 0 is described as:

(5) $$T\left(x,\;y,\;z,\;t\right)\left|{}_{t=0}\right.=T_{0}\left(x,\;y,\;z\right)$$

## 3. Modeling of Concrete and FRP Bars at High Temperatures

### 3.1. Concrete

The concrete damaged plasticity (CDP) model provided in ABAQUS 6.14 [66] software (Hibbitt, Karlsson & Sorensen, Inc., Pawtucket, RI, USA) was used as a theoretical framework to model the behavior and failure of concrete at ambient and high temperatures. The CDP model adopts the concepts of isotropic damage elasticity and isotropic tensile and compressive plasticity to represent the inelastic behavior of concrete. The theoretical framework of the CDP model includes the accurate definitions of the damage variable, the yield surface (i.e., criterion), the hardening/softening rule, and the flow potential function. The yield surface used to describe the constitutive law of concrete was originally proposed by Lubliner et al. [67] and later modified by Lee and Fenves [68], which is defined as a function of the effective stress tensor ($\bar{\sigma }$) and equivalent tensile and compressive plastic strains (${\tilde{\varepsilon }}_{t}^{pl}$ and ${\tilde{\varepsilon }}_{c}^{pl}$) as follows:

(6) $$F\left(\bar{\sigma },\;{\tilde{\varepsilon }}_{t}^{pl},\;{\tilde{\varepsilon }}_{c}^{pl}\right)\;=\frac{1}{1-\alpha }\left(\alpha I_{1}+\sqrt{3J_{2}}+\beta \langle {\bar{\sigma }}_{\max}\rangle -\gamma \langle -{\bar{\sigma }}_{\max}\rangle \right)-{\bar{\sigma }}_{c}\left({\tilde{\varepsilon }}_{c}^{pl}\right)$$

where $I_{1}$ is the first stress invariant; $J_{2}$ is the second deviatoric stress invariant; ${\bar{\sigma }}_{\max}$ and ${\bar{\sigma }}_{c}$ are the algebraic maximum eigenvalue and uniaxial compression of $\bar{\sigma }$; and   is the Macaulay bracket, $\langle x\rangle =\left(x+\left|x\right|\right)/2$. The dimensionless material constants α and β in Equation (6) can be calculated based on the uniaxial compressive yield stress $f_{c0,\;T}$, equibiaxial compressive yield stress $f_{b0,\;T}$ and uniaxial tensile yield stress $f_{t\;0,\;T}$. More details of the calculation formulas for α and β at high temperatures can be found in Gao et al. [61]. Interestingly, as the high temperature increases, the yield surface is changed from a nearly oval shape to an “egg-shape” due to the fact that the uniaxial compressive strength decreases faster than the biaxial compressive strength at high temperatures. The parameter γ is not required except for the concrete under a triaxial compression loading, i.e., the stress state of ${\bar{\sigma }}_{\max}$ < 0. The expression of γ is defined as a function of $K_{c}$ as follows:

(7) $$\gamma =\frac{3\left(1-K_{c}\right)}{2K_{c}-1}$$

where $K_{c}$ represents the ratio of the second stress invariant on the tensile meridian to that on the compressive meridian and is determined as 2/3 by default. The non-associated flow rule is used to define the flow potential function G in the CDP model, which is expressed by the following Drucker–Prager hyperbolic function:

(8) $$G=\sqrt{{\left(f_{t0,\;T}\tan\psi \epsilon \right)}^{2}+3J_{2}}+\frac{I_{1}\tan\psi }{3}$$

In the above equation, *ψ* is the dilation angle and *ϵ* is the flow potential eccentricity. In this study, *ψ* = 36° is determined based on an initial trial-and-error analysis using the proposed FE model to obtain excellent consistency with the fire test results. *ϵ* = 0.1 is determined as the default value suggested by the ABAQUS user manner [66]. In addition, the CDP model does not include damaged variables because the fire tests of the FRP-RC flexural members simulated in this study were carried out under constant service loadings, and therefore, the loading and failure of concrete do not involve the loading/unloading process. Such a consideration was also adopted in the previous study to predict the structural responses of RC beams under fire exposure [61].

**Figure 2:**
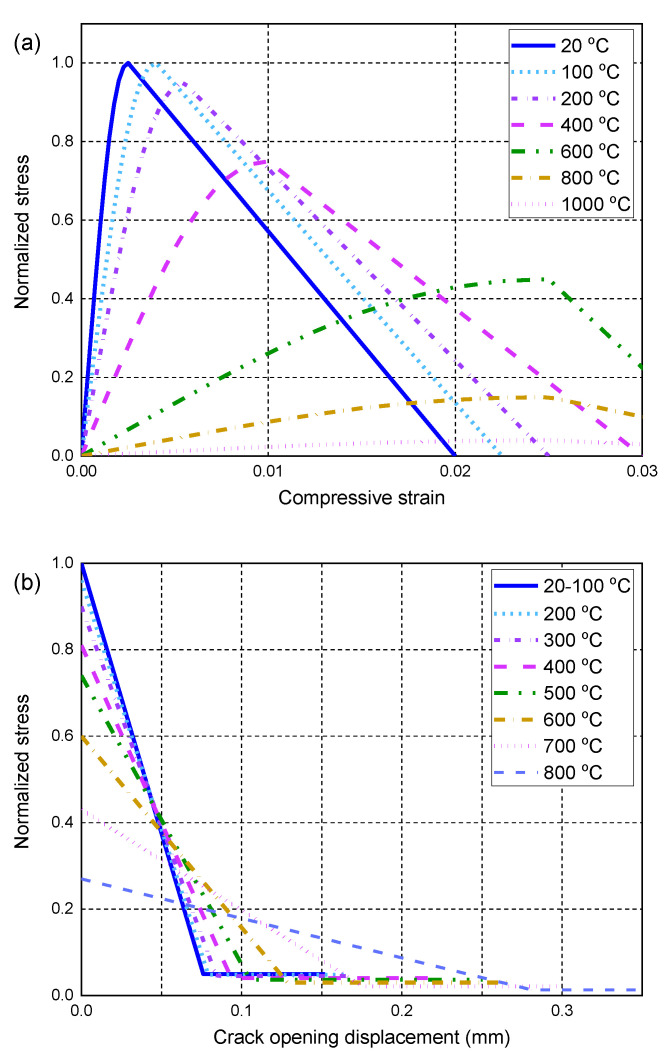
Mechanical properties of concrete at ambient and high temperatures: (**a**) normalized compressive stress–strain curves; (**b**) normalized tensile stress–crack opening displacement curves.

The hardening/softening rule controls the subsequent evolution of the yield surface and failure mode of concrete at ambient and high temperatures, which is related to the uniaxial compressive and tensile stress–strain curves of concrete. Under uniaxial compression at ambient and high temperatures, concrete behavior is defined according to the stress–strain relationship specified in EN 1992-1-2 [62]. It is worth noting that the initial stress–strain relationship is considered to be linear elastic when the compressive stresses at each temperature are less than 0.33 times the corresponding compressive strength of the concrete. Figure 2a shows the compressive stress–strain curves of concrete at ambient and high temperatures, where the high-temperature stresses are normalized by the ambient-temperature compressive strength. The tensile behavior of concrete is assumed to be elastic before cracking occurs. Concrete cracks are simulated by using a smeared crack approach in combination with the crack band model in which the tensile stress within the crack band gradually decreases with the cracking opening displacement [61]. In other words, post-peak stress behavior is defined by the softening branch (i.e., tensile softening behavior), which is described as the tensile stress versus the cracking opening displacement curve at each temperature (see Figure 2b for more details). In addition, the tensile stresses at different temperatures are normalized by the tensile strength at ambient temperature for a clear presentation. It is noteworthy that the area enclosed by the normalized tensile stress versus the cracking opening displacement curve is proportional to the fracture energy of concrete, which is assumed to be constant at ambient and high temperatures [61]. In addition, the curve of the tensile stress versus the crack opening displacement is used in the FE model instead of the tensile stress–strain curve to achieve a mesh-insensitive solution, as explained in detail in the numerical study of RC beams under fire conditions conducted by the corresponding author [61]. When the cracking opening displacement is larger than the calculated displacement corresponding to the zero tensile stress at each temperature, a residual tensile stress of 5% tensile strength is also considered to avoid possible difficulty in achieving numerical stability.

### 3.2. FRP Bars

**Figure 3:**
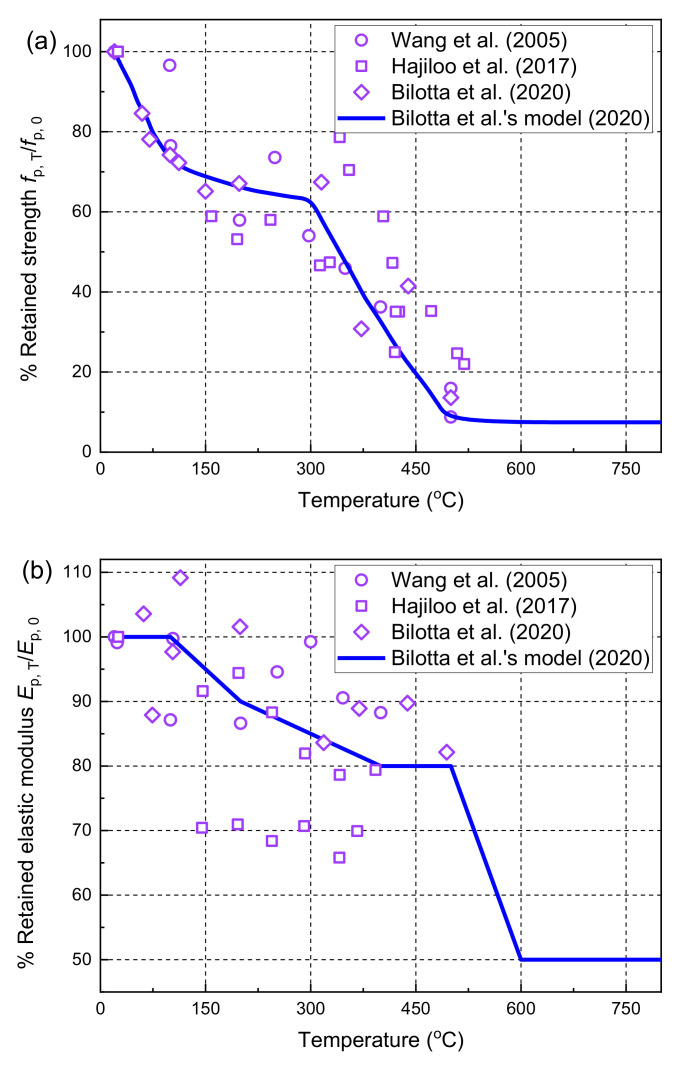
Retained mechanical properties of GFRP bars at high temperatures: (**a**) tensile strength; (**b**) elastic modulus [49,69,70].

Some tensile tests were carried out on FRP bars at ambient and high temperatures, and the results showed that the mechanical properties such as the tensile strength and elastic modulus of FRP bars decreased at moderately high temperatures [49,69,70]. The tensile stress–strain curves measured at different temperatures were almost elastic before the tensile rupture of the FRP bars. The reductions of the mechanical properties of the FRP bars were affected by the type of FRP bar and the polymer matrix, the volume ratio of the FRP fibers, and the curing conditions. Figure 3 collects the experimental data of the previous tensile tests of GFRP bars in the literature [49,69,70], and the measured results show large dispersion with the temperature changes. In the present study, the material models of GFRP bars proposed by Bilotta et al. [49] are used to describe the tensile strength and stiffness degradations at high temperatures since all fire tests of FRP-RC flexural members simulated by the FE model for validation are made of GFRP bars. As shown in Figure 3, the two rapid reduction processes at around 100 and 400 °C are related to the glass transition and decomposition processes of the polymer matrix, as explained by Reid et al. [71]. It should be noted that more tensile tests are needed to investigate the mechanical property degradations of FRP bars at high temperatures, and more accurate material models are needed to accurately describe the reductions in the tensile strength and elastic modulus at high temperatures.

## 4. Modeling of the Bond Behavior at High Temperatures

The bond interaction between the FRP bar and the surrounding concrete governs the load transfer between the two materials, which is usually described by the local bond stress–slip relationship at the FRP bar-to-concrete interface. Due to the bond action, the FRP bars located between two adjacent concrete cracks have a stiffness contribution to the tensile behavior of the concrete between the two cracks. This phenomenon is termed the “tension-stiffening” effect [72,73], and it significantly influences the deformation behavior of the FRP-RC flexural member, especially in the post-cracking stage. Such a tension-stiffening effect can be appropriately reflected by accurately simulating the bond–slip behavior of the FRP bar-to-concrete interface of the FRP-RC flexural member under bending loads. In the literature, some analytical studies were carried out to derive the local bond stress–slip behavior of the FRP bar-to-concrete interface at ambient temperature [74,75,76]. Among them, the Bertero–Popov–Eligehausen (BPE) model, originally proposed by Eligehausen et al. [77] for describing the local bond–slip relationship of deformed steel bars, was used by Cosenza et al. [74] and Rossetti et al. [75] to describe the bond–slip behavior of FRP reinforcing bars. In addition, a modified BPE model (mBPE) was proposed in previous studies [74,75] in which a two-branch model without a peak bond stress plateau was adopted. The effect of the surface treatment of FRP bars was considered by both the BPE and mBPE models, but the effects of the fiber type and bar diameter were ignored. Cosenza et al. [76] proposed a new model (i.e., the Cosenza–Manfredi–Realfonzo (CMR) model) to better describe the ascending branch of the bond–slip relationship.

At high temperatures, some pull-out tests on FRP bars have indicated that the decreases in the bond strength are more severe than the corresponding reductions in the tensile strengths of the same FRP bars [11,12,58]. In other words, the bond behavior of the FRP bar-to-concrete interface at high temperatures is the most crucial factor that governs the fire performance of FRP-RC flexural members [78]. Moreover, the loss of the bond action between FRP bars and concrete at high temperatures may lead to pull-out failure (i.e., anchorage failure) of FRP bars, resulting in a possible sudden collapse of the FRP-RC member in a fire. However, there is very limited information about the local bond–slip model that can be used to describe the bond behavior of FRP reinforcing bars at high temperatures [79]. Only Aslani [79] proposed a local bond–slip relationship to describe the interfacial behavior of GFRP bars in concrete at high temperatures. In the proposed model, the effects of some design parameters including the concrete compressive strength, bar diameter, embedment length, and concrete cover depth were considered. The accuracy of the proposed bond–slip model was only validated by a limited database of bond–slip curves measured by the pull-out tests. However, due to the lack of information available in the literature, the bond–slip model proposed by Aslani [79] is still used in the FE model to define the local constitutive law of the GFRP bar-to-concrete interface at high temperatures. Once more test data are available, it will be necessary to develop a more accurate bond–slip model that can be incorporated into the proposed FE model to more precisely describe the interfacial bond behavior at high temperatures. The bond–slip model proposed by Aslani [79] is based on the mBPE model, which consists of two branches, namely, the ascending branch before attaining the peak bond stress and the softening branch. The two branches of the local bond–slip model are expressed by the following equations:

(9) $$\begin{array}{cc} \frac{\tau_{s,\;T}}{\tau_{\max,\;T}}=\;{\left(\frac{s}{s_{\max,\;T}}\right)}^{\alpha } & \;\;\;\;\;s\leq s_{\max,\;T} \end{array}$$

(10) $$\begin{array}{cc} \frac{\tau_{s,\;T}}{\tau_{\max,\;T}}=1-\frac{p\left(s-s_{\max,\;T}\right)}{s_{\max,\;T}} & \;\;\;s>s_{\max,\;T} \end{array}$$

where $\tau_{s,\;T}$ is the bond stress at temperature T; s is the local interfacial slip; α is a curve-fitting parameter with the value less than 1; $\tau_{\max,\;T}$ is the peak bond stress at temperature T; $s_{\max,\;T}$ is the slip corresponding to the peak bond stress $\tau_{\max,\;T}$; and p is determined based on the curve-fitting of test data. $\tau_{\max,\;T}$ and $s_{\max,\;T}$ are computed by the following equations (i.e., Equations (11)–(14)).

(11) $$\frac{\tau_{\max,T}}{\tau_{\max,0}}=\left\{\begin{array}{ll} 1 & T=20\;{}^{\circ}C\\ 1.26-0.0063\times T+7\times 10^{-6}\cdot T^{2} & 20\;{}^{\circ}C<T\leq 350\;{}^{\circ}C \end{array}\right.$$

(12) $$\frac{\tau_{\max,T}}{\tau_{\max,0}}=\left\{\begin{array}{ll} 1 & T=20\;{}^{\circ}C\\ 0.989-0.00025\times T-3\times 10^{-6}\cdot T^{2} & 20\;{}^{\circ}C<T\leq 350\;{}^{\circ}C \end{array}\right.$$

(13) $$\frac{s_{\max,T}}{s_{\max,0}}=\left\{\begin{array}{ll} 1 & 20\;{}^{\circ}C\\ 0.9739-0.0019\times T-3\times 10^{-5}\cdot T^{2} & 20\;{}^{\circ}C<T\leq 350\;{}^{\circ}C \end{array}\right.$$

(14) $$\frac{s_{\max,T}}{s_{\max,0}}=\left\{\begin{array}{ll} 1 & 20\;{}^{\circ}C\\ 0.9219-0.001\times T-5\times 10^{-6}\cdot T^{2} & 20\;{}^{\circ}C<T\leq 350\;{}^{\circ}C \end{array}\right.$$

In above equations, $\tau_{\max,\;T}$ and $s_{\max,\;T}$ have two different expressions depending on the tensile strength of GFRP bars at ambient temperature (*f*_pm,0_). That is, Equations (11) and (13) are used for the cases of 500 ≤ *f*_pm,0_ ≤ 1000 MPa, while Equations (12) and (14) are adopted for the cases of 1000 < *f*_pm,0_ ≤ 1500 MPa. The parameter $\tau_{\max,0}$ in Equations (11) and (12) can be calculated as follows:

(15) $$\tau_{\max,\;0}=\left[0.55{\left(\frac{c}{d_{b}}\right)}^{0.6}+3\frac{d_{b}}{l_{d}}\right]{\left(f_{\mathrm{cm},\;0}\right)}^{0.52}$$

where $f_{\mathrm{cm},\;0}$ is the concrete compressive strength at ambient temperature (MPa); $d_{b}$ is the diameter of the GFRP bar (mm); and $l_{d}$ is the embedment length (mm); c is the concrete cover which is determined as the smaller one of the centroid of the bar to the nearest surface or the half of the center-on-center spacing of the GFRP bars (mm). $s_{\max,0}$ can be computed by Equation (16), which is a modified version of Baena et al.’s [80] model.

(16) $$s_{\max,0}=m_{0}e^{\left(m_{1}d_{b}\right)}$$

where $m_{0}$ and $m_{1}$ are two curve-fitting parameters, which are determined as 0.01 and 0.291, respectively [79].

## 5. Element Type, Interface, and Boundary Conditions

In the FE model, four parts, i.e., the concrete section, FRP bars and loading plates, and the supporting plates of the FRP-RC flexural member, need to be simulated. In the heat transfer analysis, the eight-node linear heat transfer solid element with a temperature degree of freedom (DC3D8) and the two-node heat transfer link element (DC1D2) provided by the ABAQUS software were used to model the concrete section and the GFRP bars, respectively. In other words, the loading plates and the supporting plates were not modeled in the heat transfer analysis. For the mechanical analysis, the eight-node solid element with reduced integration and hourglass control (C3D8R) was adopted to model the concrete section, the loading plates and the supporting plates, while the two-node linear 3D beam element (B33) was used in modeling the GFRP rebars. The nonlinear spring element was used to simulate the bond–slip behavior along the two tangential directions of the FRP bar-to-concrete interface in the mechanical analysis. For the normal behavior of the spring elements, the interaction between the GFRP bar and the concrete cover was defined by setting a large stiffness almost equal to the elastic stiffness of the concrete. The mesh size of all the solid elements was determined as 50 × 50 × 25 mm based on the convergence study, and the corresponding element size was adopted for the beam and spring elements. It should be noted that the configurations of all the nodes and elements between the heat transfer analysis and the mechanical analysis are the same. Therefore, the results of the cross-sectional temperature distributions generated by the heat transfer analysis can be used as an initial condition that can be properly applied to the corresponding time step of the mechanical analysis.

The loading plates were tied to the top surface of the flexural member, and the service loads acting on the member during the fire exposure were applied to the reference points on the loading plates. The supporting plates were attached (tied) to the bottom of the flexural member to ensure that there was no slip at the interface between the supporting plates and the concrete surface during the loading and fire process. The pinned boundary conditions were assigned at the reference points at the bottom of the supporting plates during the entire fire test.

## 6. Results and Discussion

**Figure 4:**
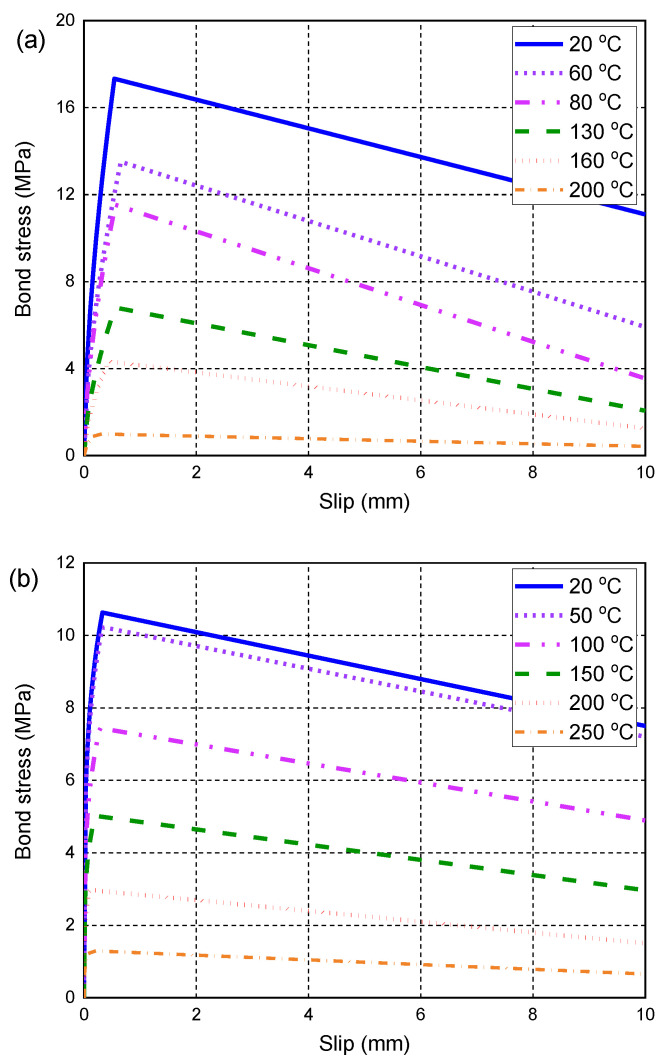
Local bond–slip model used in the FE model: (**a**) Slab GA; (**b**) Slabs S1, S2, and S3.

Two series of fire tests conducted by Hajiloo et al. [42] and Nigro et al. [43] were selected to validate the proposed FE model. The reason for choosing these fire tests for validation is that all the material properties required to define the constitutive models of GFRP bars and concrete at high temperatures are reported in detail in their original studies. In addition, the procedure of fire tests in the previous studies is well reported by the authors. Table 1 summarizes the geometric and material properties of the tested specimens. In this table, the bond properties of S1–S3, including the peak bond stress, $\tau_{\max,0}$, and the interfacial slip, $s_{\max,0}$, are computed by the local bond–slip model proposed by Aslani [79], as explained in Section 4. Moreover, Hajiloo and Green [78] conducted a series of pullout tests to determine the temperature-dependent bond behavior of the GFRP bars used in the slab GA, which generated more accurate values of the bond strengths at ambient and high temperatures. Therefore, the measured values of $\tau_{\max,\;T}$ based on the pull-out tests [78] are directly used here to define the bond–slip behavior of the slab GA, while the interfacial slip $s_{\max,\;T}$ is still calculated by the local bond–slip model proposed by Aslani [79]. It is worth noting that the calculation of $s_{\max,0}$ needs an embedded length $l_{d}$, which is determined as 5 $d_{b}$ according to previous research [70,78] if the length of the GFRP bars used in the fire test is higher than 5 $d_{b}$. Figure 4a,b depict the temperature-dependent bond–slip relationships that are incorporated into the proposed FE model for modeling the fire tests conducted by Hajiloo et al. [14] and Nigro et al. [15], respectively. In addition, the coefficients of thermal expansion of the GFRP bars and the concrete are considered to be $\alpha_{c}$ = 7.5 × 10^−6^/°C and $\alpha_{p}$ = 7.0 × 10^−6^/°C, which are determined according the suggestion provided by Hajiloo et al. [47].

### 6.1. Hajiloo et al.’s Tests

**Figure 5:**
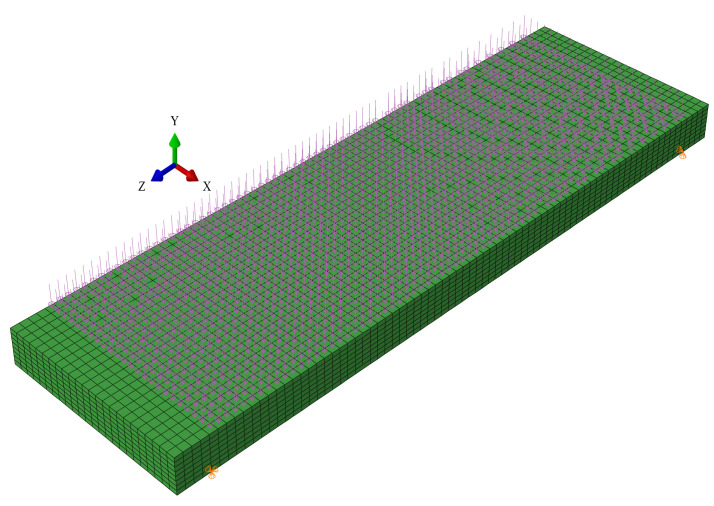
The details of the created 3D FE model of the slab GA.

Hajiloo et al. [42] conducted fire tests on two full-scale GFRP bar-reinforced concrete slabs exposed to the ASTM E119 standard fire. The concrete cover depth of the slabs was set as 60 mm to ensure that a fire-resistance rating of 2 h could be achieved as per the current design guideline of Canada [6]. The geometric and material properties of the two slabs were the same except for the different surface treatment methods used for the GFRP bars (i.e., GA denoted the sand-coated GFRP bars while GB denoted the helically wrapped GFRP bars with the sand coating). A uniformly distributed load of 19.1 kN/m was applied on the top surface of each slab, which induced a bending moment of 45 kN·m at the midspan section. In this paper, only the fire test results of the slab GA are used to validate the proposed FE model since similar test results of the two slabs are reported in the original study [42]. As shown in Table 1, the slab GA has a total length of 3900 mm, a width of 1200 mm, and a thickness of 200 mm. During the fire test, the central 3500 mm span is exposed to the fire, while the two ends of the slab are simply supported on the furnace walls with a 200 mm length at each end unexposed to fire. Figure 5 shows the mesh configuration, uniformly distributed loads, and simply-supported boundary conditions of the slab GA that are adopted in the FE simulation.

#### 6.1.1. Temperature Responses

**Figure 6:**
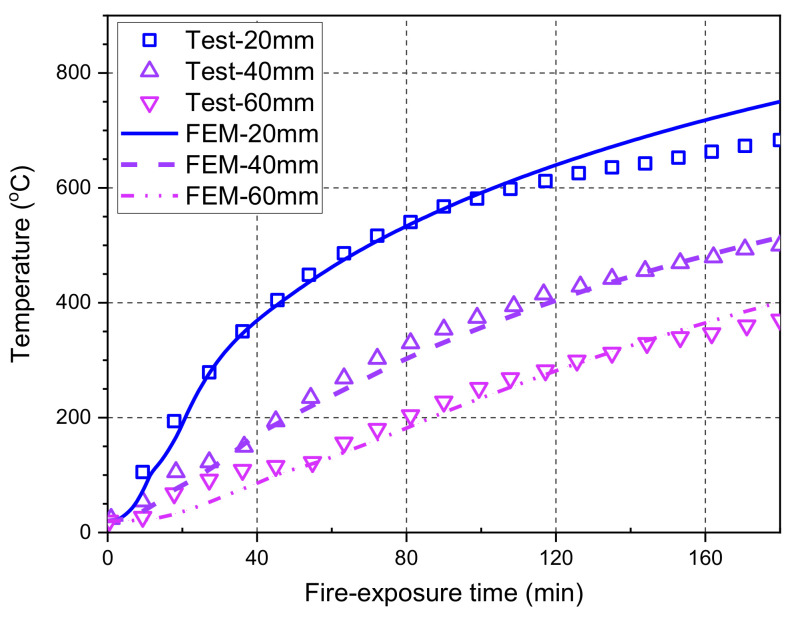
Temperature comparisons between the FE predictions and the measured results.

Figure 6 compares the predicted temperatures at different concrete depths and the corresponding measured results during the entire fire test. It can be seen from the figure that the temperatures measured at a concrete depth of 60 mm exhibit a temperature plateau at around 100 °C, possibly due to the migration and evaporation of moisture during the heating process. Therefore, the FE predictions underestimate the test results slightly in the first 40 min of fire exposure. However, such temperature underestimation has little impact on the fire resistance evaluation of the GFRP bar-reinforced concrete slab due to the almost unchanged mechanical properties of concrete and GFRP bars at relatively low temperatures (e.g., at around 100 °C). During the entire fire test, the maximum deviation of FE predictions is observed at a concrete depth of 20 mm, which is about 10% lower than the measured results. This deviation is acceptable for the temperature distribution analysis in a practical fire resistance design of structural components. Overall, the FE predictions agree well with the test data, indicating that the heat transfer analysis in the proposed FE model is accurate and reliable.

#### 6.1.2. Structural Performance

**Figure 7:**
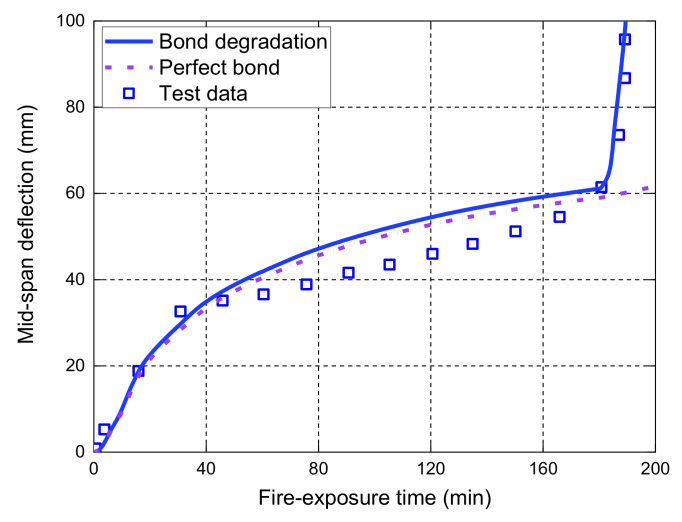
Measured deflections versus FE predictions of slab GA.

Figure 7 shows the midspan deflection responses of the slab as a function of the fire-exposure time. It should be noted that the main difference between the current FE model and the one proposed by Hajiloo et al. [47] is that the local bond–slip behavior at high temperatures is considered in our model, while a perfect bond assumption was adopted in Hajiloo et al.’s [47] study. The comparisons in Figure 7 show that the FE predictions of the midspan deflection responses are consistent with the results recorded in the fire test. Moreover, it is observed that the FE predictions of the midspan deflection responses with the local bond–slip consideration are slightly higher than those with perfect bond consideration until the tested slab is close to the failure under fire exposure. The main reason is that the load level applied on the slab during the fire test is relatively low, which yields the maximum tensile stress of about 160 MPa in the GFRP bars (i.e., only 10% of the corresponding tensile strength of the GFRP bars at ambient temperature) [42]. Since the deflection measured after 3-h fire exposure was small, the applied loads were gradually increased from 19.1 kN/m to 23.2 kN/m and the measured deflections grew quickly until the anchorage failure of the GFRP bars (i.e., the observed partial pull-out failure of the GFRP bars at the anchorage zone through a concrete cutting saw after fire test [42]). The FE predictions with the bond degradation consideration can predict the anchorage failure with an abrupt increase in the deflection responses, which are consistent with the measured results. However, the perfect bond consideration cannot give accurate predictions of the deflection responses when the applied loads are increased, demonstrating that a reliable temperature-dependent bond–slip model is needed for the deflection responses and the fire resistance evaluation of FRP-RC flexural members, especially when they are under moderate or high service load levels.

#### 6.1.3. Local Interfacial Slip Responses

**Figure 8:**
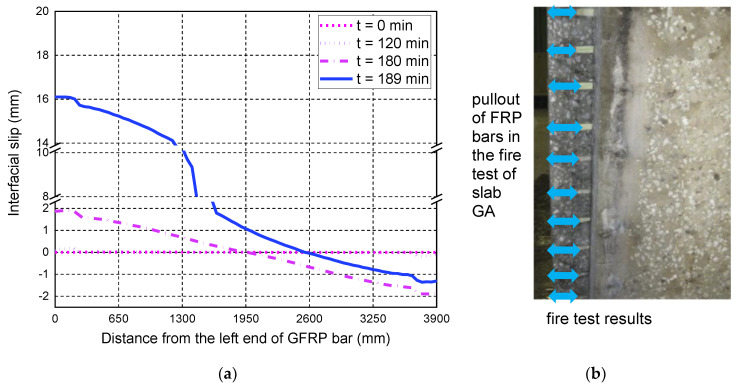
Interfacial slip distributions along the length of the GFRP bar: (**a**) FE predictions; (**b**) the observed pullout failure mode of GFRP bars.

The advantage of the proposed FE model is that it can reflect the local behavior of the GFRP bar-to-concrete interface at high temperatures, which is usually not easily measured with instruments during the fire test. Such local behavior is beneficial for explaining the anchorage failure mode of GFRP bars in the tested slab. Figure 8 shows the interfacial slip distributions along the length of the middle tensile GFRP bar at various fire-exposure times. In the initial time before the fire test (i.e., t = 0 min), the slips are almost anti-symmetrically distributed along the whole length of the GFRP bar, and the values of the applied load-induced interfacial slips are very small. As expected, the interfacial slips continue to increase with the fire-exposure time, mainly due to the high-temperature effects on the bond behavior of the FRP-to-concrete interface. In fact, the high temperatures during fire test have two different effects on the interfacial slip distributions, i.e., those caused by the interfacial thermal stresses and the bond degradations at high temperatures. The former is induced by the different thermal expansions of the GFRP bar and the surrounding concrete, while the latter is the load-induced slips due to the bond degradations. As seen in Figure 8, before the fire exposure time of 120 min, the largest interfacial slip occurs in the transition zone between the exposed span and the unexposed part, mainly due to the contribution of the thermal stress-induced slips. As the fire-exposure time increases, the local interfacial slips induced by the applied loads are increased rapidly and become the dominant effect. After 180 min of fire exposure, the maximum slippage occurs in the end anchorage zone of the GFRP bar, which eventually leads to the anchorage failure of the tested slab under fire exposure. The predicted failure mode of the tested slab is consistent with the observation of the fire test, which further indicates that the local bond–slip behavior of the GFRP bar-to-concrete interface at high temperatures needs to be considered in order to achieve an accurate prediction of the anchorage failure mode of the FRP-RC flexural member under fire exposure.

### 6.2. Nigro et al.’s Tests

Nigro et al. [43] carried out fire tests on six GFRP bar-reinforced concrete slabs exposed to the ISO 834 standard fire. The slabs were divided into two groups according to the concrete cover depth and the embedment length of the GFRP bars in the unexposed zone, and therefore, each group included three slabs. For the first group (i.e., slabs S1, S2, and S3), the concrete cover depth and the embedment length were set as 32 mm and 250 mm, respectively, which were lower than those of the second group (i.e., 51 mm and 500 mm of slabs S4, S5, and S6). The proposed FE model simulated only the first group of specimens to validate its reliability and accuracy because it suffered from more severe fire damage due to the relatively smaller concrete cover depth. Among the three slabs, S1 was loaded under its self-weight without an external load under fire exposure, while S2 and S3 were loaded with different load levels corresponding to 40% and 60% of the room-temperature flexural capacity, respectively. The geometric dimensions and reinforcement details of S1, S2, and S3 are shown in Table 1. All slabs have a length of 3500 mm, a width of 1250 mm, and a thickness of 180 mm. During the fire test, each slab was placed on the furnace walls, and the two ends of the slab could be rotated freely to achieve the simply-supported boundary conditions. The layout of the longitudinal GFRP bars at the bottom of S3 was Φ12 mm @ 225 mm, i.e., much smaller than those of S1 and S2 (i.e., Φ12 mm @ 150 mm). The slabs S2 and S3 were both loaded under four-point bending, and the two concentrated loads were symmetrically distributed at 700 mm apart from the midspan, which were both equal to 17.5 kN and were kept constant during the fire test.

#### 6.2.1. Temperature Responses

**Figure 9:**
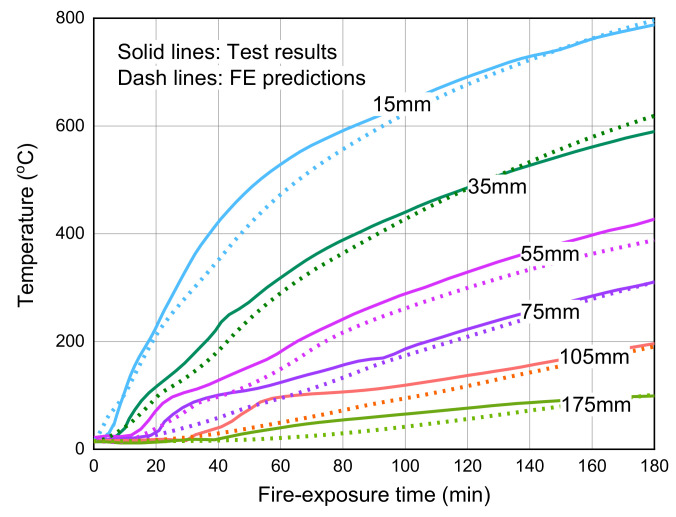
FE predictions versus measured results at various concrete depths.

Figure 9 compares the temperature responses measured at different concrete depths with the corresponding FE predictions, indicating that the heat transfer analysis of the FE model can achieve good accuracy in the temperature predictions of FRP-RC flexural members under fire exposure. The temperature responses measured in the 55 to 175 mm depths show an apparent plateau near 100 °C due to the moisture migration and evaporation. However, this moisture effect has not been considered in the proposed heat transfer analysis, and therefore, the temperature predictions are slightly lower than the measured results at the early stage of the fire test. As mentioned earlier, this temperature underestimation at relatively low temperatures (i.e., around 100 °C) is not expected to affect the mechanical analysis of the FRP-RC flexural member under fire exposure and the related fire resistance evaluation. In addition, it is observed that the temperature responses in the fire test are slightly higher than the FE predictions with the maximum deviation of about 10% at the 55 mm concrete depth. Overall, the FE predictions of all concrete depths are very similar to the test results during the entire fire exposure period.

#### 6.2.2. Structural Performance

**Figure 10:**
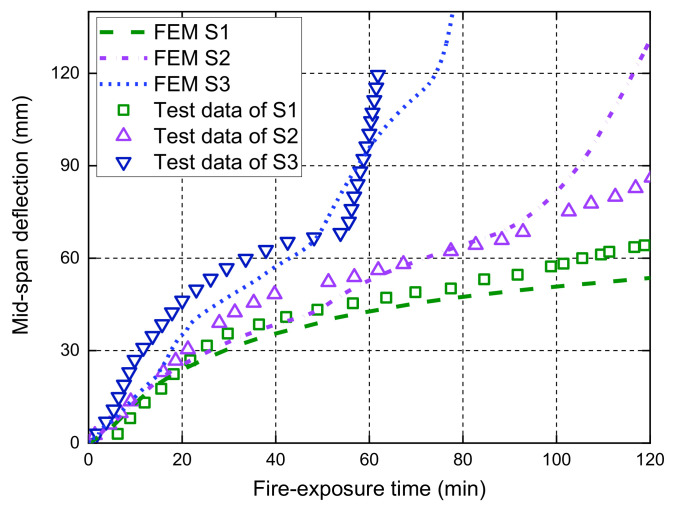
Measured deflection responses versus FE predictions of slabs S1, S2, and S3.

**Figure 11:**
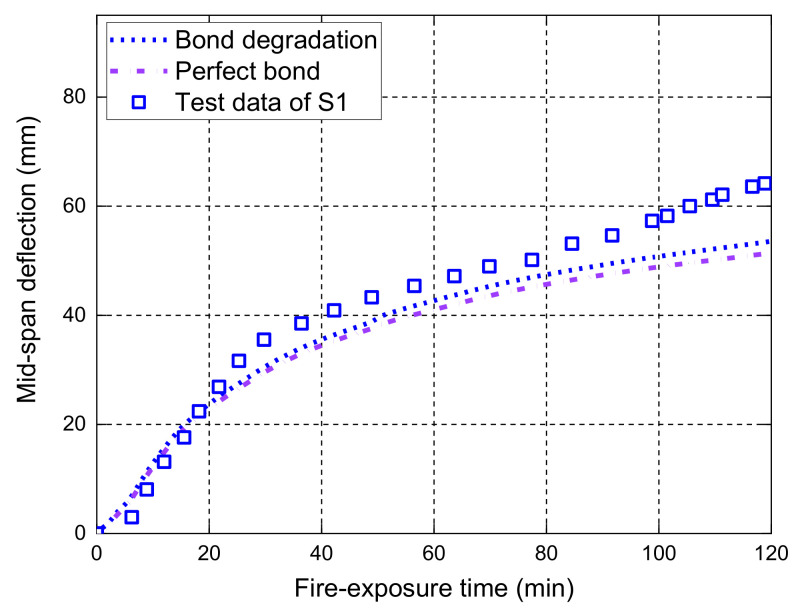
Effect of bond degradation on the midspan deflection responses of S1.

**Figure 12:**
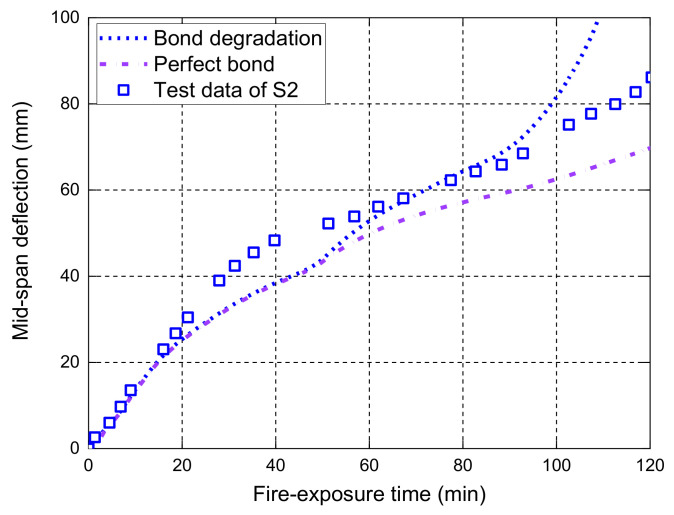
Effect of bond degradation on the midspan deflection responses of S2.

**Figure 13:**
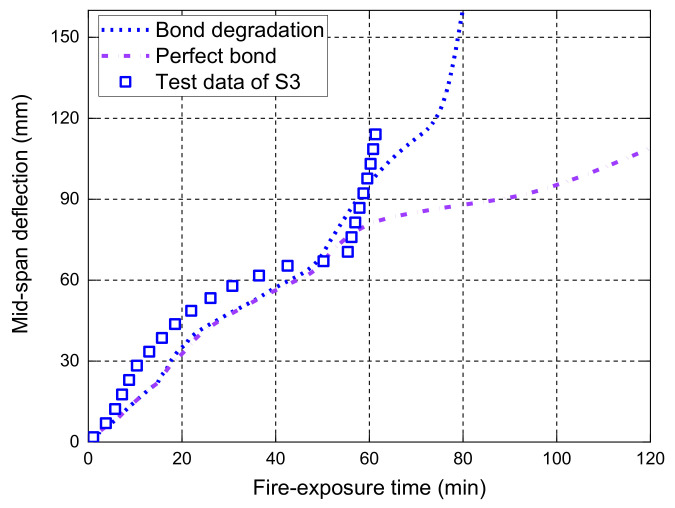
Effect of bond degradation on the midspan deflection responses of S3.

**Figure 14:**
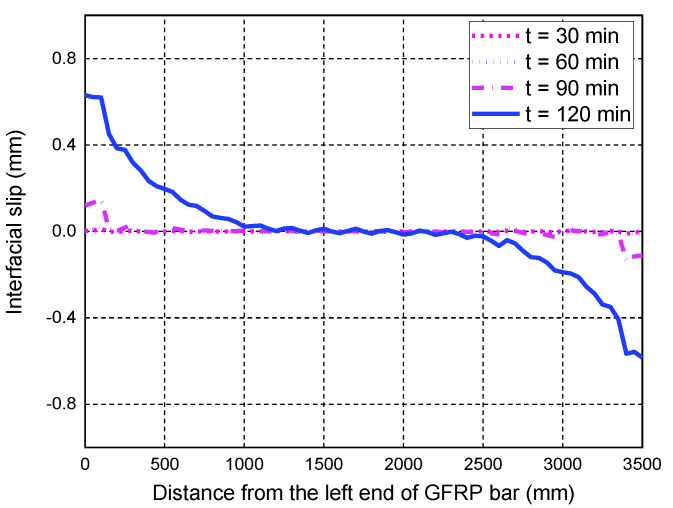
Interfacial slip distributions along the GFRP bar length of S1.

The midspan deflection responses of the slabs S1, S2, and S3 measured during the fire tests are compared with the FE predictions in Figure 10. Figure 11, Figure 12 and Figure 13 further illustrate the effects of different bond considerations on the midspan deflection responses. The comparisons between the FE predictions and the test results have indicated that the FE model with the bond degradation consideration achieves reasonably good consistency, although the predicted fire resistance period of S3 is about 80 min, which is larger than the fire test result. In addition, the FE model with the perfect bond consideration often produces less accurate predictions. For, slab S1, the midspan deflections predicted by the FE model with the degraded bond consideration are slightly higher than those with perfect bond consideration. This is because no external load is applied on the slab during fire exposure, and thus the stress level of the GFRP bar is very low. This observation is confirmed by the interfacial slip distributions along the GFRP bar length at various fire-exposure times as shown in Figure 14. In this figure, the maximum interfacial slip obtained during the entire fire test is small and less than 0.8 mm. Therefore, for the specific slab under the self-weight loading, the different bond considerations have little effect on the midspan deflection responses.

#### 6.2.3. Local Interfacial Slip Responses

**Figure 15:**
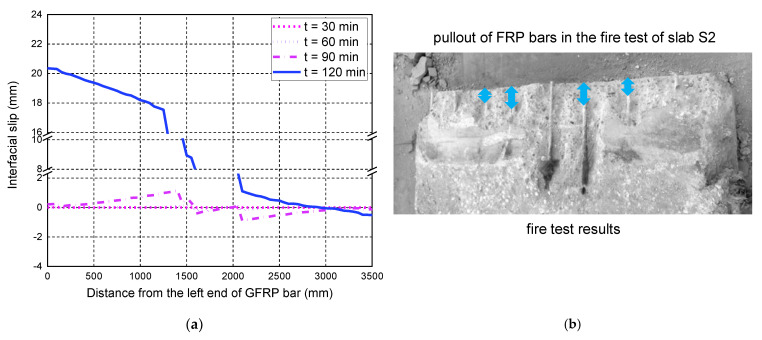
Interfacial slip distributions along the GFRP bar length of S2: (**a**) FE predictions; (**b**) the observed pullout failure mode of GFRP bars.

**Figure 16:**
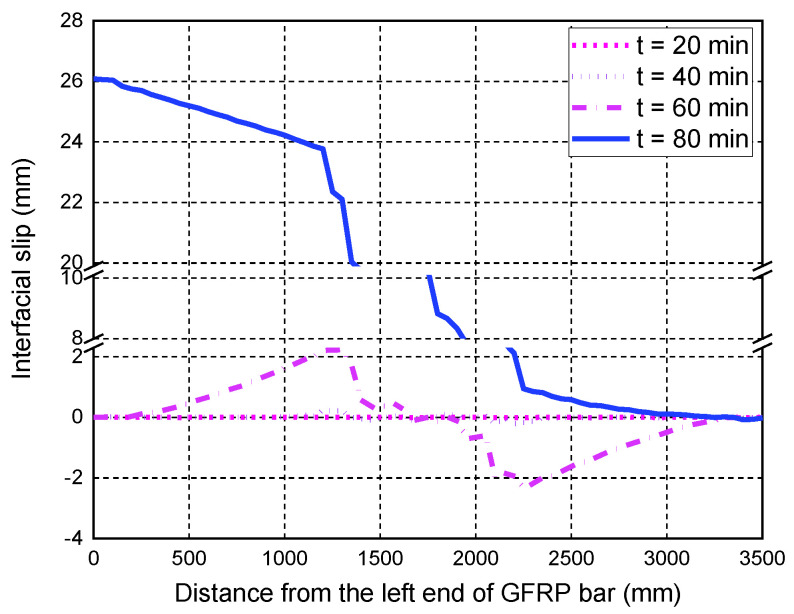
Interfacial slip distributions along the GFRP bar length of S3.

For slab S2, the consideration of temperature-dependent bond degradations has a significant impact on the deflection responses, mainly due to the larger applied load of S2 compared with S1. In the fire test, it was observed that two main cracks appeared at the bottom of slab S2, located below the two loading points. The FE predictions of the interfacial slips in Figure 15 show that the maximum slips after 90 min of fire exposure occur at the positions below the loading points, mainly induced by the main concrete cracks. Therefore, the consistency between the test observation and the local slip predictions has further proved the reliability of the proposed FE model. Slab S3 eventually failed due to the anchorage failure of the GFRP bars, which is also confirmed by the predicted interfacial slip responses at 120 min. The FE predictions of the slips in the end anchorage zone are quite large (i.e., about 20 mm), indicating the pull-out failure of the GFRP bar in the anchorage zone. Since S3 has a relatively low reinforcement ratio, the load level corresponding to the room-temperature flexural capacity is much higher than that of S2. Therefore, the influence of different bond degradations on the deflection responses during the fire exposure is more significant. Once again, the FE model with the bond degradation consideration can accurately predict the midspan deflection responses during fire exposure and the associated anchorage failure of slab S3, as shown in Figure 13 and Figure 16.

## 7. Conclusions

This paper has proposed a 3D FE model to predict the fire performance of FRP-RC flexural members with appropriate considerations of the constitutive models of FRP bars, concrete, and their bond interface at high temperatures. The latter issue has not been accurately simulated by the previous numerical studies. The proposed FE model has been validated by comparing the FE results with the test data of the full-scale fire tests of FRP-RC slabs in the literature. The following conclusions can be drawn based on the results presented in the paper:

(1) The proposed FE model has good accuracy in predicting the thermal and structural responses of FRP-RC flexural members under fire exposure. The constitutive models used to define the thermal and mechanical properties of concrete and FRP bars at high temperatures are reliable. The temperature predictions of the proposed FE model are very similar to the measured results, with a maximum deviation of about 10% during the entire fire test.
(2) The perfect bond consideration of the FRP bar-to-concrete interface at high temperatures often yields less accurate predictions of midspan deflection responses. Thus, proper consideration of the local bond–slip behavior of the FRP bar-to-concrete interface at high temperatures is required for accurately predicting the midspan deflection responses of FRP-RC flexural members exposed to fire.
(3) The consideration of the local bond–slip behavior also gives detailed information on the interfacial slip responses at the FRP bar-to-concrete interface at high temperatures during a fire, which can reveal the concrete cracking pattern and failure mode of the tested member under fire exposure. The predicted maximum interfacial slips of the FRP bars near the end of the fire test are almost 20 mm.
(4) The proposed FE model can accurately predict the anchorage failure of FRP bars in the FRP-RC flexural member during fire exposure, which is a typical failure mode in the existing fire tests in the literature. The previous numerical studies based on a perfect bond consideration cannot provide a reliable prediction for this failure mode.

Further research is needed to develop more reliable constitutive models of FRP bars and bond interface at high temperatures, which are necessary for more accurate predictions of the deflection responses of the FRP-RC flexural members under fire exposure.

## Figures and Tables

**Table 1:** Geometric and material properties of the tested specimens.

Specimen ^a^	Geometry ^b^ (mm)	GFRP Bar (mm)	Applied Load	Material Property ^c^	Bond Property
Slab GA	*l* = 3900 *b* = 1200 *h* = 200 *c* = 68 *l*_n_ = 3840 *l*_f_ = 3500	Tensile: Φ16@100 (longitudinal) Φ13@200 (transverse) Compressive: Φ13@220 (longitudinal) Φ13@200 (transverse)	19.1 kN/m	*f*_c,0_ = 28.9 MPa *f*_t,0_ = 2.8 MPa *f*_p,0_ = 1500 MPa *E*_p,0_ = 66 GPa	*τ*_max,0_ = 17.33 MPa *s*_max,0_ = 0.81 mm
Slab S1	*l* = 3500 *b* = 1250 *h* = 180 *c* = 32 *l*_n_ = 3200 *l*_f_ = 3000	Tensile: Φ12@150 (longitudinal) Φ12@200 (transverse) Compressive: Φ12@200 (longitudinal) Φ12@200 (transverse)	None (Self-weight)	*f*_c,0_ = 38.6 MPa *f*_t,0_ = 3.2 MPa *f*_p,0_ = 1000 MPa *E*_p,0_ = 50 GPa	*τ*_max,0_ = 10.63 MPa *s*_max,0_ = 0.33 mm
Slab S2		Tensile: Φ12@150 (longitudinal) Φ12@200 (transverse) Compressive: Φ12@200 (longitudinal) Φ12@200 (transverse)	2 × 17.5 kN		
Slab S3		Tensile: Φ12@225 (longitudinal) Φ12@200 (transverse) Compressive: Φ12@200 (longitudinal) Φ12@200 (transverse)	2 × 17.5 kN		

Notes: ^a^ Slab GA was tested by Hajiloo et al. [42], while the slabs S1, S2, S3 were taken from Nigro et al. [43]. ^b^ *l*—total length, *b*—slab width, *h*—slab height, *c*—concrete cover (defined as the distance from the slab bottom to the centroid of the GFRP bars), *l*_n_—slab span, *l*_f_—fire-exposed span. ^c^ *f*_c,0_ and *f*_t,0_—compressive strength and tensile strength of concrete at ambient temperature; *f*_p,0_ and *E*_p,0_—tensile strength and elastic modulus of GFRP bars at ambient temperature.

## Data Availability

All data, models, and code generated or used during the study appear in the published article.

## References

[1] Bakis C.E., Bank L.C., Asce F., Brown V.L., Asce M., Cosenza E., Davalos J.F., Asce A.M., Lesko J.J., Machida A. (2002). Fiber-Reinforced Polymer Composites for Construction—State-of-the-Art Review. J. Compos. Constr..

[2] Gravina R.J., Li J., Smith S.T., Visintin P. (2020). Environmental Durability of FRP Bar-to-Concrete Bond: Critical Review. J. Compos. Constr..

[3] Bellakehal H., Zaidi A., Masmoudi R., Bouhicha M. (2014). Behavior of FRP Bars-Reinforced Concrete Slabs under Temperature and Sustained Load Effects. Polymers.

[4] Ghahari S.A., Assi L.N., Alsalman A., Alyamaç K.E. (2020). Fracture Properties Evaluation of Cellulose Nanocrystals Cement Paste. Materials.

[5] ACI (American Concrete Institute) (2015). 440.1R-15, Guide for the Design and Construction of Concrete Reinforced with FRP Bars.

[6] CSA (Canadian Standards Association) (2012). CSA-S806, Design and Construction of Building Components with Fiber-Reinforced Polymers.

[7] Wang Y., Ren Z., Huang Z., Gao W., Zhong B., Bu Y., Huang Y., Zhang Y., Yuan G., Ma S. (2021). Experimental and numerical studies of six small-scale continuous concrete slabs subjected to travelling fires. Eng. Struct..

[8] Song J., Gao W.-Y., Ouyang L.-J., Zeng J.-J., Yang J., Liu W.-D. (2021). Compressive behavior of heat-damaged square concrete prisms confined with basalt fiber-reinforced polymer jackets. Eng. Struct..

[9] Ouyang L.-J., Wei X.-X., Ding B., Gao W.-Y. (2021). Effective Strain of BFRP for Confined Heat-Damaged Concrete Cylinders. Front. Mater..

[10] Ouyang L.-J., Chai M.-X., Song J., Hu L.-L., Gao W.-Y. (2021). Repair of thermally damaged concrete cylinders with basalt fiber-reinforced polymer jackets. J. Build. Eng..

[11] Katz A., Berman N., Bank L. (1999). Effect of High Temperature on Bond Strength of FRP Rebars. J. Compos. Constr..

[12] Solyom S., Di Benedetti M., Guadagnini M., Balázs G.L. (2020). Effect of temperature on the bond behaviour of GFRP bars in concrete. Compos. Part B Eng..

[13] Hajiloo H., Green M.F. (2018). Post-fire residual properties of GFRP reinforced concrete slabs: A holistic investigation. Compos. Struct..

[14] Rafi M.M., Nadjai A., Ali F. (2008). Finite Element Modeling of Carbon Fiber-Reinforced Polymer Reinforced Concrete Beams under Elevated Temperatures. ACI Struct. J..

[15] Wang K., Young B., Smith S. (2011). Mechanical properties of pultruded carbon fibre-reinforced polymer (CFRP) plates at elevated temperatures. Eng. Struct..

[16] Bai Y.-L., Yan Z.-W., Ozbakkaloglu T., Gao W.-Y., Zeng J.-J. (2021). Mechanical behavior of large-rupture-strain (LRS) polyethylene naphthalene fiber bundles at different strain rates and temperatures. Constr. Build. Mater..

[17] Dai J.-G., Gao W.-Y., Teng J.G. (2013). Bond–slip Model for FRP Laminates Externally Bonded to Concrete at Elevated Temperature. J. Compos. Constr..

[18] Silva M.A., Biscaia H. (2008). Degradation of bond between FRP and RC beams. Compos. Struct..

[19] Gao W.-Y., Teng J.G., Dai J.-G. (2012). Effect of Temperature Variation on the Full-Range Behavior of FRP-to-Concrete Bonded Joints. J. Compos. Constr..

[20] Wilson J., Gao W.-Y., Yang J. (2020). Effect of temperature variations on interfacial debonding of FRP-plated beams: A coupled mix-mode cohesive zone modeling. IOP Conf. Ser. Mater. Sci. Eng..

[21] Leone M., Matthys S., Aiello M.A. (2009). Effect of elevated service temperature on bond between FRP EBR systems and concrete. Compos. Part B Eng..

[22] Firmo J., Correia J., Pitta D., Tiago C., Arruda M. (2015). Experimental characterization of the bond between externally bonded reinforcement (EBR) CFRP strips and concrete at elevated temperatures. Cem. Concr. Compos..

[23] Guo D., Gao W.-Y., Fernando D., Dai J.-G. (2022). Effect of temperature variation on the plate-end debonding of FRP-strengthened beams: A theoretical study. Adv. Struct. Eng..

[24] Jia D.-G., Gao W.-Y., Duan D.-X., Yang J., Dai J.-G. (2021). Full-range behavior of FRP-to-concrete bonded joints subjected to combined effects of loading and temperature variation. Eng. Fract. Mech..

[25] Gao W.-Y., Dai J.-G., Teng J. (2015). Analysis of Mode II debonding behavior of fiber-reinforced polymer-to-substrate bonded joints subjected to combined thermal and mechanical loading. Eng. Fract. Mech..

[26] Guo D., Gao W.-Y., Dai J.-G. (2022). Effects of temperature variation on intermediate crack-induced debonding and stress intensity factor in FRP-retrofitted cracked steel beams: An analytical study. Compos. Struct..

[27] Joshani M., Koloor S., Abdullah R. (2012). Damage Mechanics Model for Fracture Process of Steel-Concrete Composite Slabs. Appl. Mech. Mater..

[28] Hawileh R., Naser M.Z., Abdalla J.A. (2013). Finite element simulation of reinforced concrete beams externally strengthened with short-length CFRP plates. Compos. Part B Eng..

[29] Gao W.-Y., Dai J.-G., Teng J.G. (2015). Simple Method for Predicting Temperatures in Insulated, FRP-Strengthened RC Members Exposed to a Standard Fire. J. Compos. Constr..

[30] Gao W.-Y., Dai J.-G., Teng J.G. (2016). Fire resistance design of un-protected FRP-strengthened RC beams. Mater. Struct..

[31] Dong K., Hu K., Gao W.-Y. (2016). Fire Behavior of Full-Scale CFRP-Strengthened RC Beams Protected with Different Insulation Systems. J. Asian Arch. Build. Eng..

[32] Firmo J., Correia J. (2015). Fire behaviour of thermally insulated RC beams strengthened with EBR-CFRP strips: Experimental study. Compos. Struct..

[33] Liu F., Wu B., Wei D. (2009). Failure modes of reinforced concrete beams strengthened with carbon fiber sheet in fire. Fire Saf. J..

[34] Gao W.-Y., Dai J.-G., Teng J.G. (2018). Three-Level Fire Resistance Design of FRP-Strengthened RC Beams. J. Compos. Constr..

[35] Gao W.-Y., Hu K.-X., Lu Z.-D. (2010). Fie Resistance Experiments of Insulated CFRP Strengthened Reinforced Concrete Beams. China Civil Eng. J..

[36] Palmieri A., Matthys S., Taerwe L. (2012). Experimental investigation on fire endurance of insulated concrete beams strengthened with near surface mounted FRP bar reinforcement. Compos. Part B Eng..

[37] Williams B., Kodur V., Green M., Bisby L. (2008). Fire Endurance of Fiber-Reinforced Polymer Strengthened Concrete T-Beams. ACI Struct. J..

[38] Zhu H., Wu G., Zhang L., Zhang J., Hui D. (2014). Experimental study on the fire resistance of RC beams strengthened with near-surface-mounted high-Tg BFRP bars. Compos. Part B Eng..

[39] Dai J.-G., Gao W.-Y., Teng J.G. (2015). Finite Element Modeling of Insulated FRP-Strengthened RC Beams Exposed to Fire. J. Compos. Constr..

[40] Ahmed A., Kodur V. (2011). Effect of bond degradation on fire resistance of FRP-strengthened reinforced concrete beams. Compos. Part B Eng..

[41] Firmo J.P., Arruda M., Correia J., Rosa I.C.M. (2018). Three-dimensional finite element modelling of the fire behaviour of insulated RC beams strengthened with EBR and NSM CFRP strips. Compos. Struct..

[42] Hajiloo H., Green M.F., Noël M., Bénichou N., Sultan M. (2017). Fire tests on full-scale FRP reinforced concrete slabs. Compos. Struct..

[43] Nigro E., Cefarelli G., Bilotta A., Manfredi G., Cosenza E. (2011). Fire resistance of concrete slabs reinforced with FRP bars. Part I: Experimental investigations on the mechanical behavior. Compos. Part B Eng..

[44] Rosa I.C., Santos P., Firmo J.P., Correia J.R. (2020). Fire behaviour of concrete slab strips reinforced with sand-coated GFRP bars. Compos. Struct..

[45] Kodur V.K., Bisby L.A., Foo S.H. (2005). Thermal Behavior of Fire-Exposed Concrete Slabs Reinforced with Fiber-Reinforced Polymer Bars. ACI Struct. J..

[46] Rafi M.M., Nadjai A. (2011). Fire Tests of Hybrid and Carbon Fiber-Reinforced Polymer Bar Reinforced Concrete Beams. ACI Mater. J..

[47] Hajiloo H., Green M.F. (2019). GFRP reinforced concrete slabs in fire: Finite element modelling. Eng. Struct..

[48] Nigro E., Cefarelli G., Bilotta A., Manfredi G., Cosenza E. (2011). Fire resistance of concrete slabs reinforced with FRP bars. Part II: Experimental results and numerical simulations on the thermal field. Compos. Part B Eng..

[49] Bilotta A., Compagnone A., Esposito L., Nigro E. (2020). Structural behaviour of FRP reinforced concrete slabs in fire. Eng. Struct..

[50] Rafi M.M., Nadjai A. (2013). Numerical modelling of carbon fibre-reinforced polymer and hybrid reinforced concrete beams in fire. Fire Mater..

[51] Nigro E., Cefarelli G., Bilotta A., Manfredi G., Cosenza E. (2014). Guidelines for flexural resistance of FRP reinforced concrete slabs and beams in fire. Compos. Part B Eng..

[52] Rafi M.M., Nadjai A. (2014). Parametric finite element analysis of FRP reinforced concrete beams in fire and design guidelines. Fire Mater..

[53] Masmoudi A., Masmoudi R., Ben Ouezdou M. (2010). Thermal effects on GFRP rebars: Experimental study and analytical analysis. Mater. Struct..

[54] Galati N., Nanni A., Dharani L.R., Focacci F., Aiello M.A. (2006). Thermal effects on bond between FRP rebars and concrete. Compos. Part A Appl. Sci. Manuf..

[55] Katz A., Berman N. (2000). Modeling the effect of high temperature on the bond of FRP reinforcing bars to concrete. Cem. Concr. Compos..

[56] Li C., Gao D., Wang Y., Tang J. (2017). Effect of high temperature on the bond performance between basalt fibre reinforced polymer (BFRP) bars and concrete. Constr. Build. Mater..

[57] Abbasi A., Hogg P.J. (2005). Temperature and environmental effects on glass fibre rebar: Modulus, strength and interfacial bond strength with concrete. Compos. Part B Eng..

[58] Hamad R.J., Johari M.M., Haddad R.H. (2017). Mechanical properties and bond characteristics of different fiber reinforced polymer rebars at elevated temperatures. Constr. Build. Mater..

[59] Nigro E., Bilotta A., Cefarelli G., Manfredi G., Cosenza E. (2012). Performance under Fire Situations of Concrete Members Reinforced with FRP Rods: Bond Models and Design Nomograms. J. Compos. Constr..

[60] Gao W.-Y., Dai J.-G., Teng J.-G. (2017). Fire resistance of RC beams under design fire exposure. Mag. Concr. Res..

[61] Gao W.-Y., Dai J.-G., Teng J., Chen G. (2013). Finite element modeling of reinforced concrete beams exposed to fire. Eng. Struct..

[62] CEN (European Committee for Standardization) (2004). Eurocode 2-Design of Concrete Structures. Part 1–2: General Rules—Structural Fire Design.

[63] Griffis C., Masumura R., Chang C. (1981). Thermal Response of Graphite Epoxy Composite Subjected to Rapid Heating. J. Compos. Mater..

[64] CEN (European Committee for Standardization) (2002). Eurocode 1-Actions on Structures. Part 1–2: General Actions—Actions on Structures Exposed to Fire.

[65] Gao W., Dai J., Teng J. (2014). Simple Method for Predicting Temperatures in Reinforced Concrete Beams Exposed to a Standard Fire. Adv. Struct. Eng..

[66] ABAQUS (2014). ABAQUS Standard User’s Manual. Version 6.14, Vol. I–III.

[67] Lubliner J., Oliver J., Oller S., Onate E. (1989). A plastic-damage model for concrete. Int. J. Solids Struct..

[68] Lee J., Fenves G.L. (1998). Plastic-Damage Model for Cyclic Loading of Concrete Structures. J. Eng. Mech..

[69] Wang Y., Kodur V. (2005). Variation of strength and stiffness of fibre reinforced polymer reinforcing bars with temperature. Cem. Concr. Compos..

[70] Hajiloo H., Green M., Gales J. (2018). Mechanical properties of GFRP reinforcing bars at high temperatures. Constr. Build. Mater..

[71] Reid E.R., Bilotta A., Bisby L.A., Nigro E. Mechanical Properties of Fibre Reinforced Polymer Reinforcement for Concrete at High Temperature. Proceedings of the 8th International Conference on Structures in Fire.

[72] Prakhya G.K.V., Morley C.T. (1990). Tension-Stiffening and Moment-Curvature Relations of Reinforced Concrete Elements. ACI Struct. J..

[73] Torres L., López-Almansa F., Bozzo L.M. (2004). Tension-Stiffening Model for Cracked Flexural Concrete Members. J. Struct. Eng..

[74] Cosenza E., Manfredi G., Realfonzo R. (1997). Behavior and Modeling of Bond of FRP Rebars to Concrete. J. Compos. Constr..

[75] Rossetti V.A., Galeota D., Giammatteo M.M. (1995). Local bond stress–slip relationships of glass fibre reinforced plastic bars embedded in concrete. Mater. Struct..

[76] Cosenza E., Manfredi G., Realfonzo R. Analytical Modelling of Bond between FRP Reinforcing Bars and Concrete. Non-Metallic (FRP) Reinforcement for Concrete Structures. Proceedings of the Second International RILEM Symposium (FRPRCS-2).

[77] Eligehausen R., Popov E.P., Bertero V.V. (1983). Local Bond Stress–Slip Relationships of Deformed Reinforcing Bars Under Generalised Excitations.

[78] Hajiloo H., Green M. (2018). Bond Strength of GFRP Reinforcing Bars at High Temperatures with Implications for Performance in Fire. J. Compos. Constr..

[79] Aslani F. (2019). Residual bond between concrete and reinforcing GFRP rebars at elevated temperatures. Proceedings of the Institution of Civil Engineers—Structures and Buildings.

[80] Baena M., Torres L., Turon A., Barris C. (2009). Experimental study of bond behaviour between concrete and FRP bars using a pull-out test. Compos. Part B Eng..

